# The New Treatment for the Tobacco Habit

**Published:** 1914-11

**Authors:** John G. Abele

**Affiliations:** Portland, Oregon


### The New Treatment for the Tobacco Habit.

BY JOHN G. ABELE, M. D., PORTLAND, OREGON.

Early this year, as most of you no doubt remember, the lay press published news items of a powerful new cure for the tobacco habit. Nitrate of silver in a mild solution, was the remedy that was working the miracle.

It happened that I had an opportunity to try it out on a couple of heavy smokers, and after a few treatments I believed it was making good. About that time the juvenile court took up the idea, and I agreed to give the treatments free of charge to the wards of the court. Only one case was sent to me by the court, and he took one treatment and never reappeared.

About then an anti-tobacco crusade was started in the public and private schools of the city, and they produced another case. He took the treatments for about four weeks, and according to his own statements is cured, but his teacher tells me that at times he simply reeks from stale tobacco smoke.

I treated fifty cases in all and expected to make nearly a perfect score at the beginning, but soon I hoped for 50 per cent, and then that went a-glimmering, and now there are none.

The apparent total failure of this much vaunted remedy I attribute to the fact that nearly every one of my cases were either coaxed to take the treatment, took it out of curiosity, or were forced to submit to it. None of them seemed to care, and I honestly believe that some quit because they were afraid it actually would break them of the habit.

One day I saw one of them throw away a butt before coming into my office, and we had a heart to heart talk. His side of it was that he had been coerced into taking the treatment, and although the silver made tobacco taste rotten, he hoped to wear out the bad taste. He has done so, and now rolls 15 cents worth of Durham into cigarettes every forty-eight hours. This was his average consumption before using the silver. During the period of treatment (one week) he never could smoke over five cigarettes in twenty-four hours.

In spite of the poor showing I have made with this remedy, I believe that it is worth trying, where you have a patient who really wants to quit. I have tried both the nitrate and nitrite, and believe the former (nitrate) the better one. I used it in a 2 per cent solution to swab the throat, or in a one-half of 1 per cent solution as a mouth wash and gargle after meals.—*Medical Sentinel.*

